# The morphology of antennal lobe projection neurons is controlled by a POU-domain transcription factor *Bmacj6* in the silkmoth *Bombyx mori*

**DOI:** 10.1038/s41598-017-14578-4

**Published:** 2017-10-25

**Authors:** Shigehiro Namiki, Tsuguru Fujii, Toru Shimada, Ryohei Kanzaki

**Affiliations:** 1 Research Center for Advanced Science and Technology, The University of Tokyo, 4-6-1 Komaba, Meguro, Tokyo, 153-8904 Japan; 2 Graduate School of Agricultural and Life Sciences, The University of Tokyo, 1-1-1 Yayoi, Bunkyo, Tokyo, 113-8567 Japan; 3 Laboratory of Silkworm Genetic Resources, Institute of Genetic Resources, Graduate School of Bio Resources and Bioenvironmental Science, Kyushu University, Fukuoka, 812-8581 Japan

## Abstract

How to wire a neural circuit is crucial for the functioning of the nervous system. Here, we describe the neuroanatomy of the olfactory neurons in the *spli* mutant strain of silkmoth (*Bombyx mori*) to investigate the function of a transcription factor involved in neuronal wiring in the central olfactory circuit. The genomic structure of the gene *Bmacj6*, which encodes a class IV POU domain transcription factor, is disrupted in the *spli* mutant. We report the neuroanatomical abnormality in the morphology of the antennal lobe projection neurons (PNs) that process the sex pheromone. In addition to the mis-targeting of dendrites and axons, we found axonal bifurcation within the PNs. These results indicate that the morphology of neurons in the pheromone processing pathway is modified by *Bmacj6*.

## Introduction

The functioning of the central nervous system depends on the proper wiring of individual neurons. The development of wiring specificity requires precise control over gene expression, which is carried out through transcription factors^1–3^. The molecular mechanisms underlying neuronal wiring have been well investigated in the olfactory system of *Drosophila melanogaster*. Genetic engineering has been very successful in *Drosophila*, and various molecular mechanisms have been identified^4^. In contrast, there are few studies about the genetic basis of neuronal wiring for other insect species.

Here, we investigate the olfactory system of the silkmoth, *Bombyx mori*, which has been used as a model for studying sex pheromone communication systems in moths. Based on comparability with *Drosophila*, we choose *B. mori* as a reference species for studying neuronal wiring^5^. As in *Drosophila*, whole genome sequencing is complete^6,7^, and a variety of genetic manipulation techniques are available in *B. mori*, including gene targeting^8^. Moths and flies share a basic set of neuroanatomical modules in the brain^9^. Furthermore, recent studies report the similarity in the morphology at the single neuron level between *B. mori* ^10,11^ and *Drosophila* ^12^.

The antennal lobe (AL), the primary olfactory centre in the insect brain, is composed of spherical structures called glomeruli. The macroglomerular complex (MGC) is a group of glomeruli, which process sex pheromones. We focused on PNs innervating the toroid glomerulus in the MGC, which processing the bombykol, the sex pheromone of *B. mori*. The projection neurons (PNs) convey the processed information in the AL to the higher order brain centre^13^. A POU-domain transcription factor, *acj6*, is known to control the dendritic targeting and axonal branching of PNs in *Drosophila* ^14^. In the *spli* mutant of *B. mori*, the genomic structure of *Bmacj6*, a homologue of the *Drosophila acj6*, is disrupted^15,16^. To examine the function of *Bmacj6* on the neuronal development of the PNs in *B. mori*, we obtained the single cell morphology of the PNs in the brains of the silkmoth *spli* mutant using intracellular staining with a sharp-glass microelectrode. Comprehensive datasets of three-dimensional morphology are available for silkmoth PNs^17–21^. Comparing normal moth strain morphology with mutant morphology, we identify the neuroanatomical abnormality of neurons in the *spli* mutant of *B. mori*.

## Results

### Uniglomerular projection neurons

**Figure 1:**
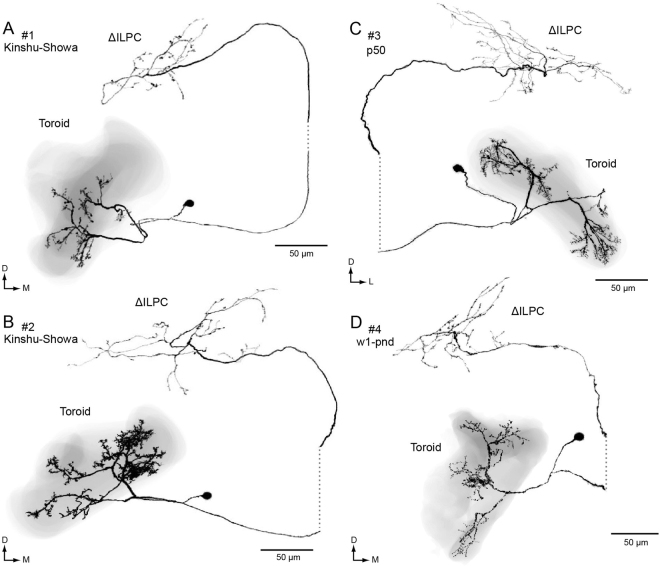
Morphology of toroid projection neurons in the normal moth strain. Neuronal morphology of PNs in strains of Kinshu-Showa (**A**,**B**), p50 (**C**) and w1-pnd are shown (**D**). The neurons are reconstructed from confocal stacks obtained with a 40× objective. The neuron is shown with black, and the shape of the toroid glomerulus is shown with transparent grey. The neurons have smooth processes within the toroid and send axon projections into the protocerebrum. The original data are taken from^10,17,22^. Two neurons also have processes with the calyx of the mushroom body (**B**,**D**). Note that no bifurcation was observed along the pathway from the antennal lobe to the protocerebrum. D, dorsal; L, lateral; M, medial; ΔILPC, the delta area of the inferior lateral protocerebrum.

Uniglomerular PNs, which have dendritic innervations onto a single glomerulus, are the major population of neuronal output from the AL in insects^13^. Figure 1 shows the representative morphology of uniglomerular PNs from normal strains of silkmoth, including Kinshu-Showa, p50 and w1-pnd. The same neuroanatomical features were observed in PNs of these strains. The dendrites are confined within the toroid glomerulus and extraglomerular innervation, the process that extends outside the glomerulus, is rare in the PNs. PNs have several groups of dendritic shafts within the glomerulus. The axons run through the AL tract, without any bifurcation. The axons project into the delta area of the inferior lateral protocerebrum (ΔILPC), the second order centre for sex-pheromone processing^20^.

**Figure 2:**
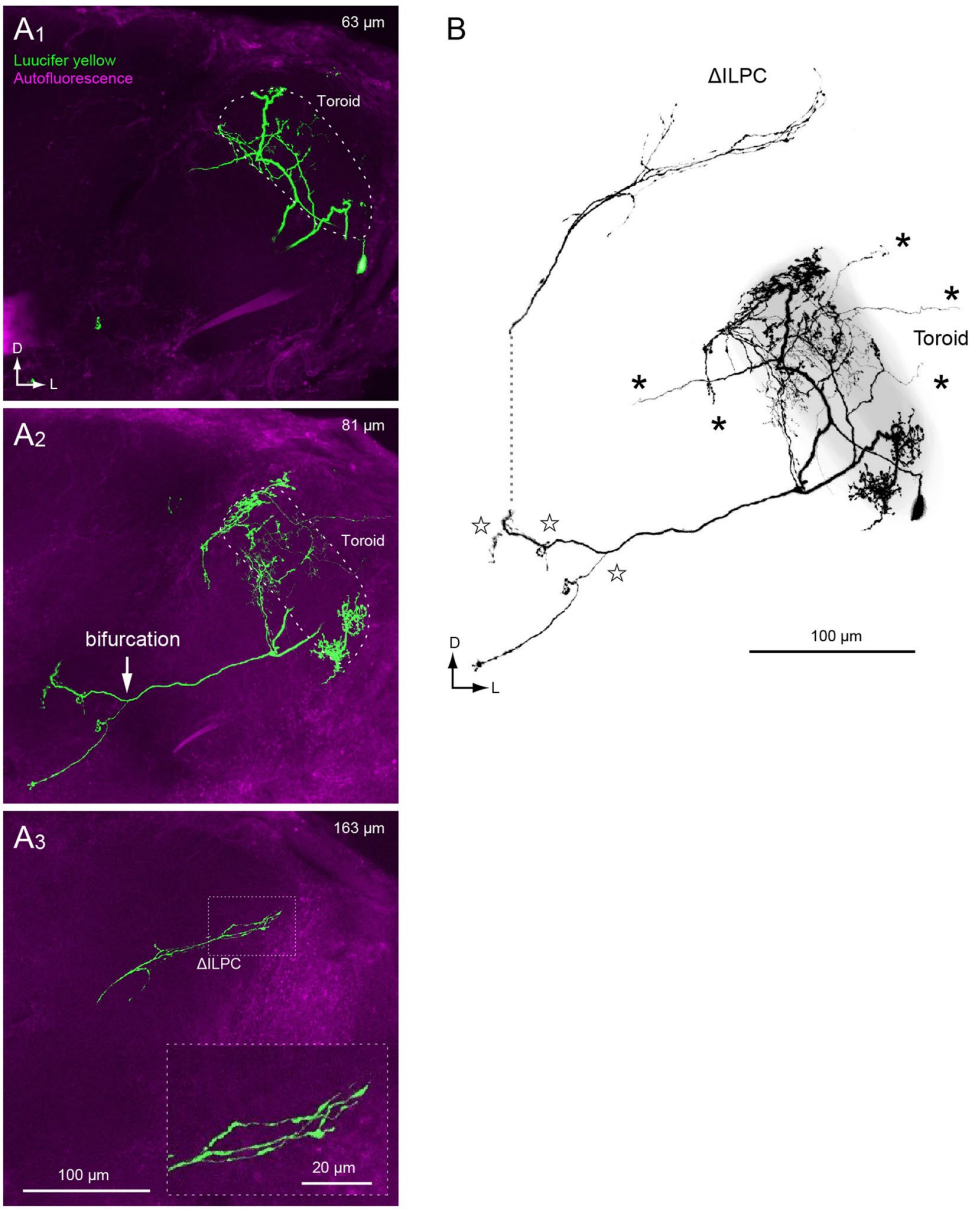
Morphology of toroid projection neurons in the *spli* mutant. (**A**) Confocal stacks of toroid PN running through the medial antennal lobe tract. The depth from the anterior surface is shown in the *top right*. The shape of the toroid glomerulus is shown with a broken line (A1, 2). There is a bifurcation of the axon projection through the antennal lobe tract (A2, arrow). The axonal projection in the protocerebrum is sparse (A3), in comparison with normal strains (Fig. 1). Inset in A3 shows a magnification of the axon terminals in the ΔILPC. The original data are taken from^16^. (**B**) Three-dimensional reconstruction of the toroid PN. The shape of the toroid is shown in grey. The processes in the AL are located mostly in the toroid, and the innervations outside the glomerulus are present (asterisks). Bifurcations are observed in several locations along with the antennal-lobe tract (stars). The axonal projection in the protocerebrum is sparse in comparison with the projection neurons in normal strains (Fig. 1).

These anatomical features observed in normal strains were altered in PNs of the *spli* mutant. Figure 2 shows an example of intracellular staining within a toroid PN. We compared this morphology with PNs from the normal strains and identified the neuroanatomical abnormality: *spli* PNs usually show extraglomerular processes. The dendrites in these mutants are not limited to the toroid and often spill out into the area outside the glomerulus (Fig. 2B, asterisks). In this example, the neuron has diffuse dendrites in some areas within the glomerulus. These are located within the cortex of the glomerulus (the area close to the glomerular surface), which is rarely observed in uniglomerular PNs, whose total number is estimated to be ~117 in the normal strain of *B. mori* ^17^.

We also observed abnormalities in the axon morphology of *spli* mutants. Axons of mutant toroid PNs always reach the ΔILPC, which is the central target of toroid PNs in the normal strain^20,21^. However, bifurcation occurs on the axon along the AL tract in the *spli* mutant (Fig. 2B, stars), which is not observed in the normal strains^17,20,21^. The terminals of bifurcated axons in the mutant strain appear varicose-like (Fig. 2B), suggesting that they may be forming a presynaptic terminal^23^. These bifurcations were observed in six out of seven examples in which axonal processes were successfully labelled. *spli* mutant axons project to roughly similar locations as the normal strain, but the density of axonal projections is often reduced in comparison with the normal strain (Fig. 2B for *spli*, Fig. 1 for normal strain, ΔILPC) (*n* = 3 out of 4 total neurons).

**Figure 3:**
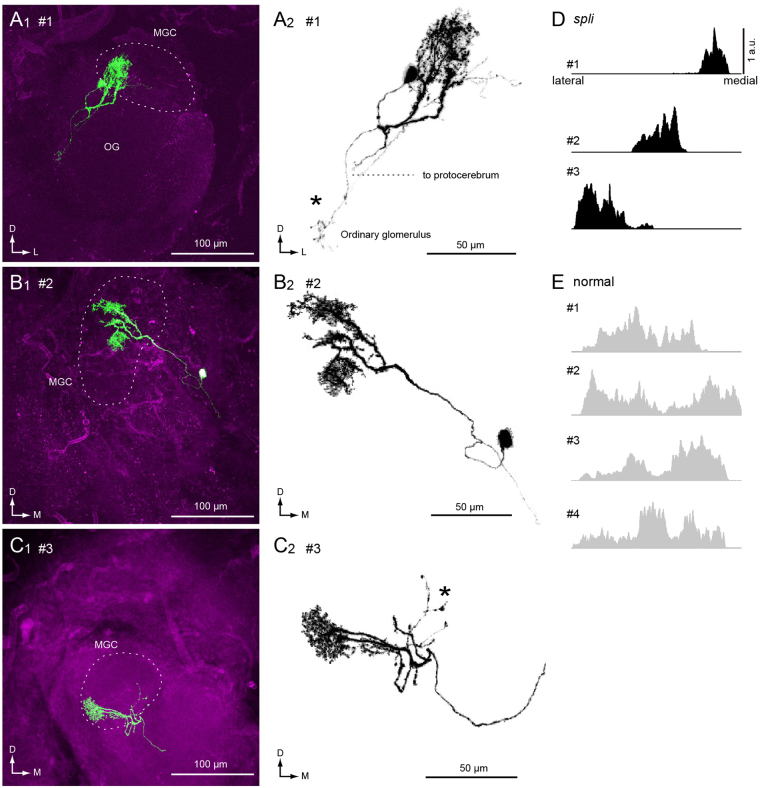
Morphology of projection neurons with a localized dendritic field in the *spli* mutant. (**A1–C1**) Maximum intensity projection of the confocal image. The broken line shows the shape of the macroglomerular complex (MGC). Three-dimensional reconstruction of the innervation in the antennal lobe (**A2–C2**). The neuron has additional processes in the one ordinary glomerulus in addition to the toroid; one of the glomeruli is in the MGC. Extraglomerular innervation is marked with an asterisk. Cell body is missing in (**C**). (**D**) Bar histogram of the dendritic volume of *spli* PNs within the MGC. The relative density normalized by the maximum value was plotted. The horizontal space is adjusted based on the axis from the lateral to medial margin of the MGC. Each bar represents the volume at the lateral position. The volume is normalized by the maximum value for each neuron. (**E**) Dendrite distribution of PNs in the normal strain. The cell ID shown in the left correspond to those used in Fig. 1.

Figure 3 shows another example of intracellular staining within PNs in the *spli* mutant. In these examples, the anatomical borders among MGC glomeruli were ambiguous; the shapes of the MGCs are shown with a broken line (Fig. 3A1–C1). The dendritic innervations are located in the MGC, which corresponds with the location of dendritic innervations of the toroid in the normal strain. The borders among glomeruli in the AL of *spli* mutants were generally more ambiguous than in the normal strain. Among 18 total ALs observed, normal arrangement of glomeruli was observed in 12 specimens. The change in glomerular size was observed in 5 specimens. The volume of toroid glomerulus was enlarged in four specimens, and that of cumulus, another pheromone glomerulus in the MGC, was enlarged in one specimen. In one case, the glomerular organization was highly disrupted.

**Figure 4:**
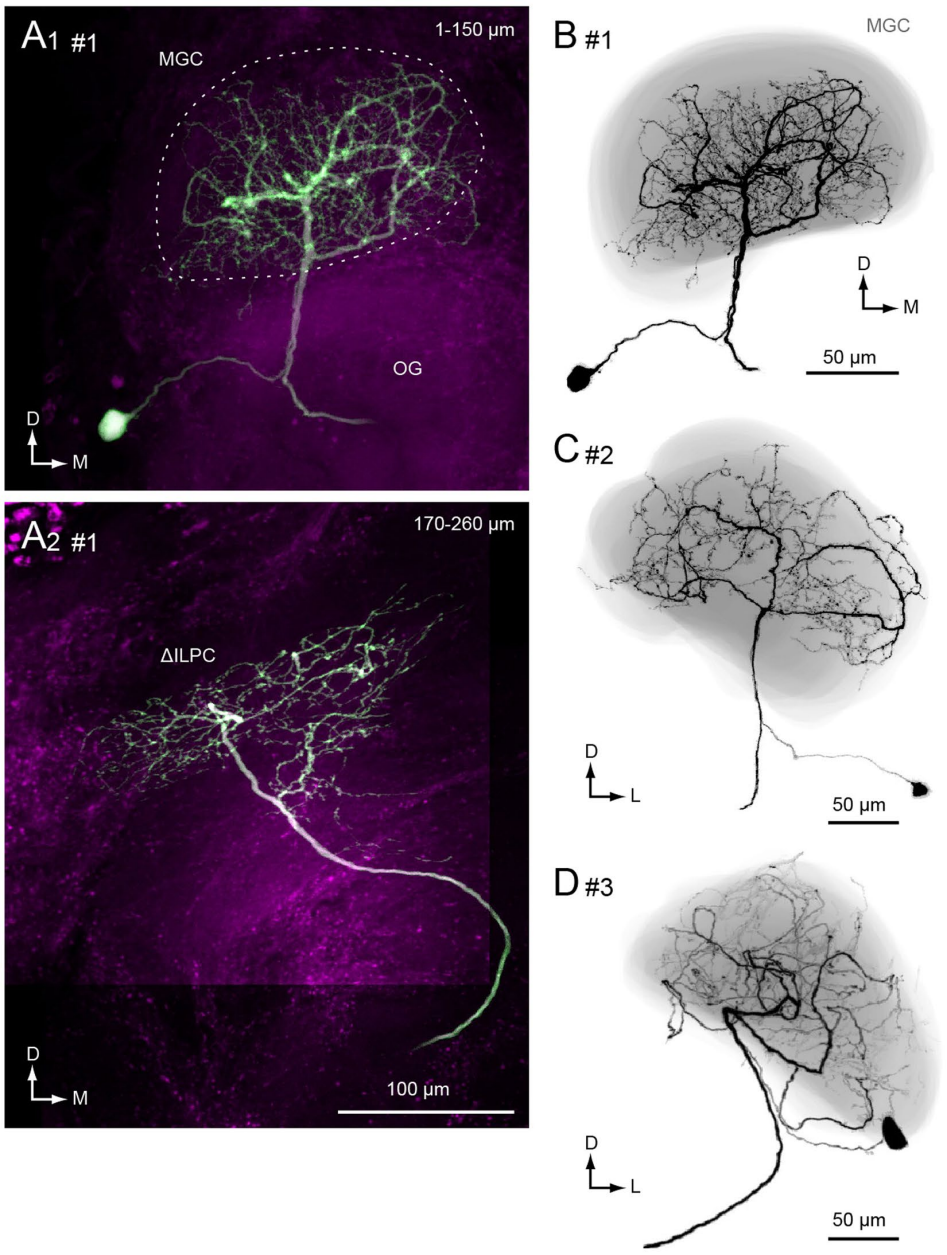
Morphology of multiglomerular projection neurons in the macroglomerular complex of the normal moth strain (Kinshu-Showa). (**A**) Maximum intensity projections for anterior and posterior halves of the brain are shown. The depth ranges are shown in the *top right*. The shape of the macroglomerular complex is shown with a broken line (MGC). The neuron has innervation in a large volume of the MGC. The original data are taken from^17^. (**B**–**D**) Three-dimensional reconstructions of multiglomerular projection neurons. The shape of the MGC is shown with grey. The neurite process is confined within the MGC.

We have previously reported the dendritic distribution of MGC PNs, and they have wide-field branches along the mediolateral axis with multiple dendritic shafts (Fig. 1) (Figs 3 and 4 in^17^). In contrast to the normal strain, the dendrites of *spli* PNs tend to be spatially localized within the MGC. Figure 3D and E show the dendritic distribution of PNs in the *spli* mutant and normal strain. The volume of the dendrite was smaller in the normal strains (*n* = 5, 44,927 ± 11,093 µm^3^) than in the *spli* mutant (*n* = 5, 14,399 ± 10,341 µm^3^; *p* = 0.0159, Mann-Whitney *U* test). In one case, we observed a PN with extraglomerular innervation, which entered into a single ordinary glomerulus outside of the MGC (Fig. 3A). The additional branches were varicose appearance, suggesting innervation into the ordinary glomerulus (Fig. 3A2, asterisk). No PNs from the normal strain innervated both the MGC and specific ordinary glomerulus simultaneously (Fig. 1)^17^, suggesting that this morphological feature is characteristic to the *spli* mutant.

### Multiglomerular projection neurons

**Figure 5:**
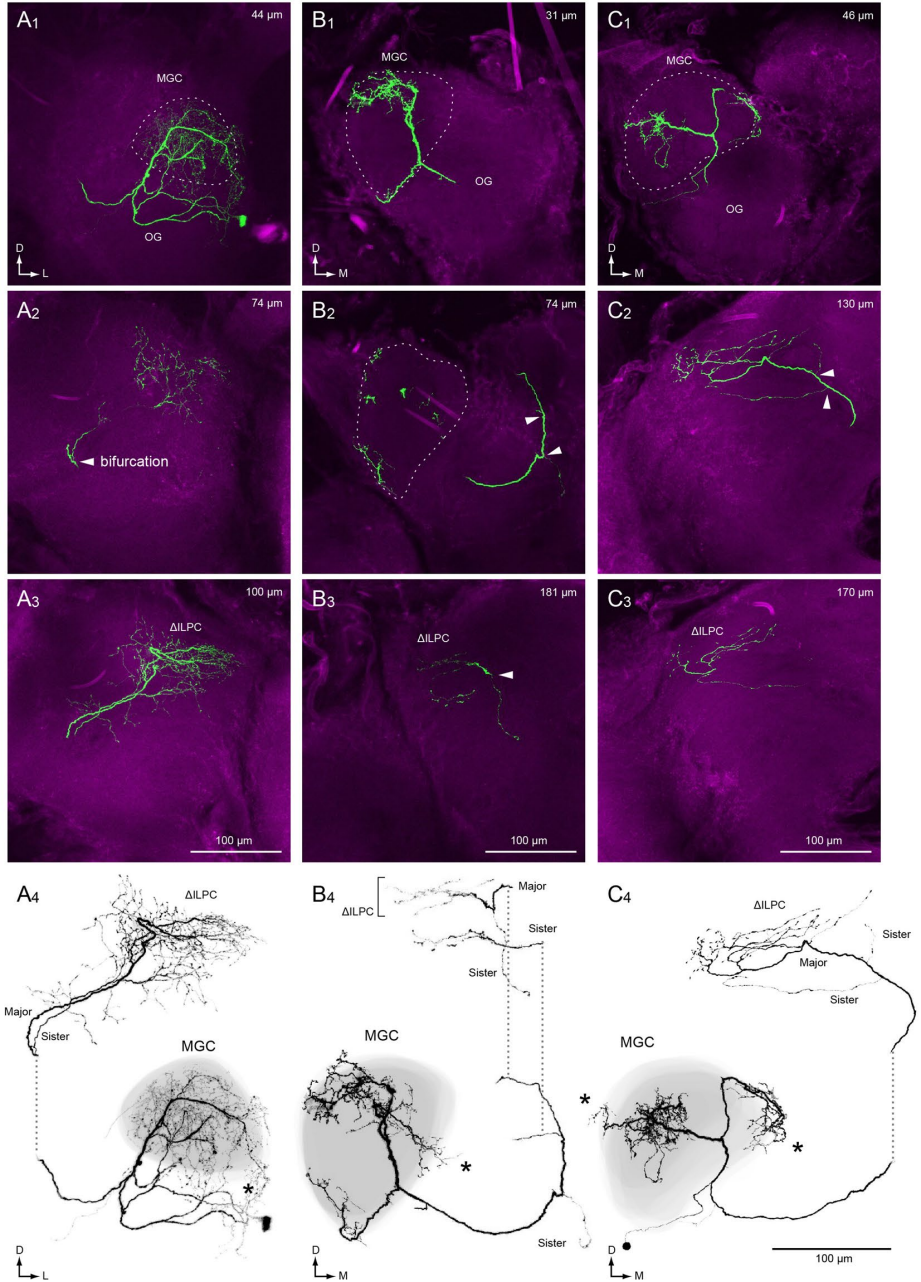
Morphology of multiglomerular projection neurons in the macroglomerular complex of the *spli* mutant. (**A**–**C**) Confocal stacks (1–3) and the maximum intensity projection throughout the neuron are shown (4). The depths from the anterior surface of the brain are shown in the *top right*. An extraglomerular process is marked with asterisk. In each sample, the bifurcation of an axon occurred. The site of bifurcation is shown with an arrowhead. The bifurcated branch with the largest diameter is labelled with ‘major’, and the others are labelled with ‘sister’. MGC, macroglomerular complex; OG, ordinary glomeruli.

Another population of AL output neurons, multiglomerular PNs, innervate multiple glomeruli^21,24^. They typically innervate both toroid and cumulus glomeruli in the MGC (Fig. 4). Multiglomerular PNs are thought to process information derived from sex pheromones and thus contribute to the recognition of species-specific pheromones from conspecifics^25^. We identified the neuronal morphology of multiglomerular PNs in the *spli* mutant in four specimens (Fig. 5). The average volume of dendritic branches was 33,292 ± 17,736 µm^3^ for PNs in the *spli* mutant (*n = *4) and 49,387 ± 15,871 µm^3^ in for PNs in normal strain (Kinshu-Showa, *n* = 5). Morphological changes in dendritic innervation were observed in all four examples. The dendritic innervation is often localized to the small area within the MGC in the *spli* mutant (Fig. 5B,C), whereas PNs innervate nearly the entire MGC in normal strain silkmoths (Fig. 4). In one mutant, the dendrites occupied the entire MGC as in the normal strain but also had dense extraglomerular innervation, most of which was smooth in appearance (Fig. 5A).

**Figure 6:**
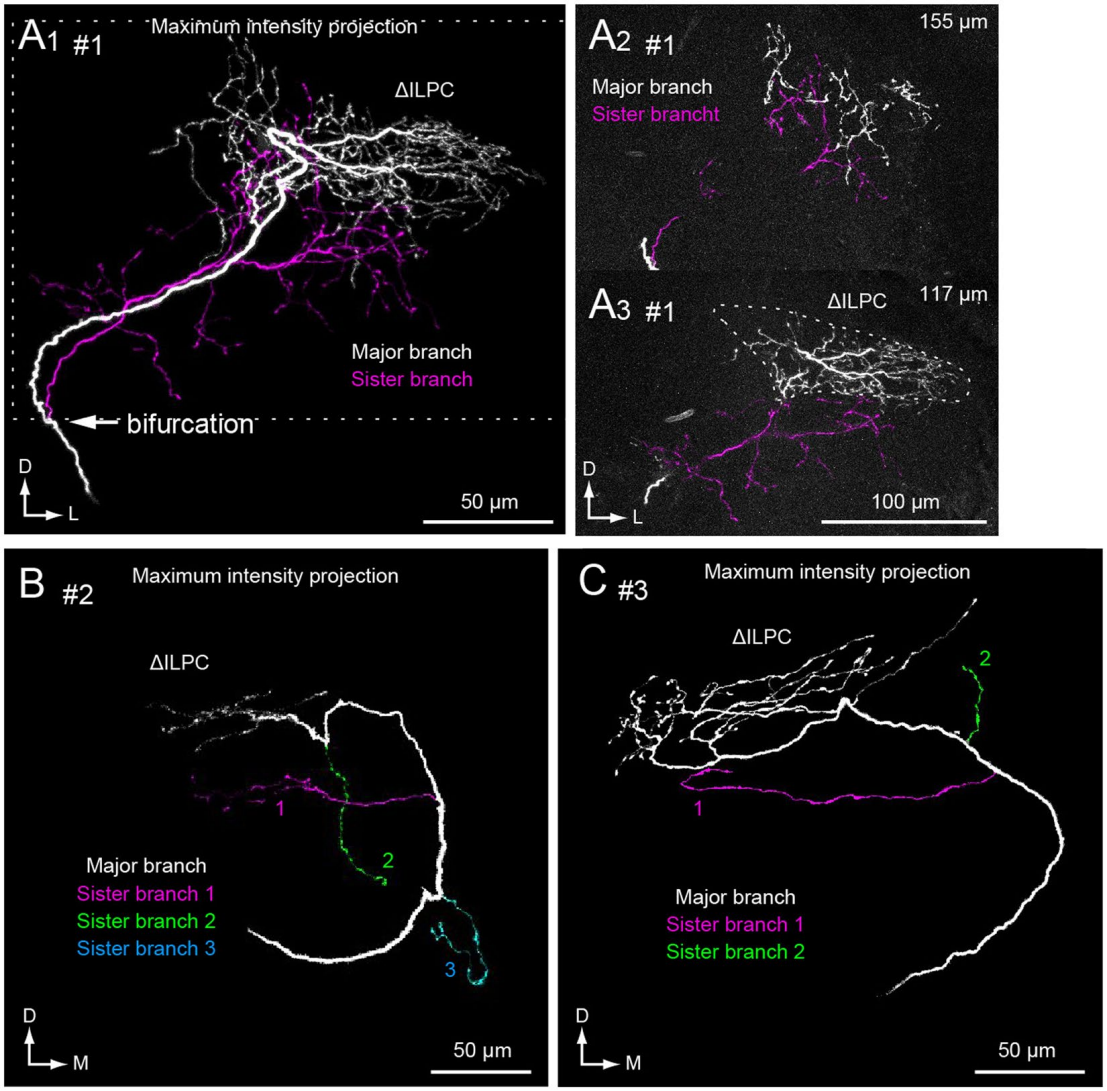
Mistargeting of a sister axon branch in the *spli* PNs. (**A**) The maximum intensity projection (A1) confocal stacks (A2,3) of a multiglomerular PN of the *spli* mutant. The depths from the posterior surface of the brain are shown in the *top right* (A2,3). The major and sister branches are shown with white and magenta. The point of bifurcation of the axon is shown with an arrow. A major axon branch projected to the delta area of the inferior lateral protocerebrum (ΔILPC), whereas terminals of sister branches were likely to project to an outside area. (**B**,**C**) The maximum intensity projection of confocal images of multiglomerular PNs. The dendrites are not shown. The major branch is shown with white, and sister branches are shown with colour. The reduction of axonal volume was observed in (**B**), and the terminals are localized towards the lateral side of the ΔILPC. The numbering of neurons corresponds to Fig. 5.

As in the uniglomerular PNs, we observed bifurcation of axonal projections in three separate examples (Fig. 5A–C). To analyse the characteristics of bifurcated axons, we segmented individual branches manually by using image processing software (Fig. 6). Among bifurcated branches, the thickest process usually projected to a similar location as the projections in the normal strain. We call this process a ‘major branch,’ and smaller processes are ‘sister branches’. In contrast to the major axonal branch, sister branches usually displayed abnormal projection patterns. Figure 6A demonstrates an example of bifurcated axon in multiglomerular PN. Although the sister branch projects in a similar direction along with the major branch after the branching point, the projection destinations are different. The terminals of the major branch are located within the ΔILPC, whereas the terminals of the sister branch are primarily located outside of the ΔILPC, including an unstructured neuropil located medioventrally to the ΔILPC (Fig. 6A). The bifurcation occurred with other types of abnormality. Mis-targeting of sister axon branches was also observed in all examples (Fig. 6B,C). Among these, the reduction of neurite volume was observed in 2 specimens (Fig. 5B,C).

### Local interneurons

The transcription factor *acj6* is expressed in specific neurons in *Drosophila*. For example, *acj6* is not expressed in local interneurons (LNs), which are intrinsic cells in the AL, and no neuroanatomical abnormalities have been reported in this cell type^26^. We successfully stained two LNs in *B. mori* (Supplementary Fig. 1). The arborisations of LNs were restricted to ordinary glomeruli, and no branches innervated the MGC. We have previously classified LNs into five different types in the normal strain based on the anatomical features^27^. The two identified LNs in the *spli* mutant were classified as a specific cell type termed the oligoGs-a LNs, whose innervations are limited to the ordinary glomeruli^27^. As in oligoGs-a LNs of the normal strain, we observed both dense (Supplementary Fig. 1B, anterior-medial to the AL) and sparse innervation of these LNs (Supplementary Fig. 1B, posterior-lateral to the AL).

## Discussion

**Figure 7:**
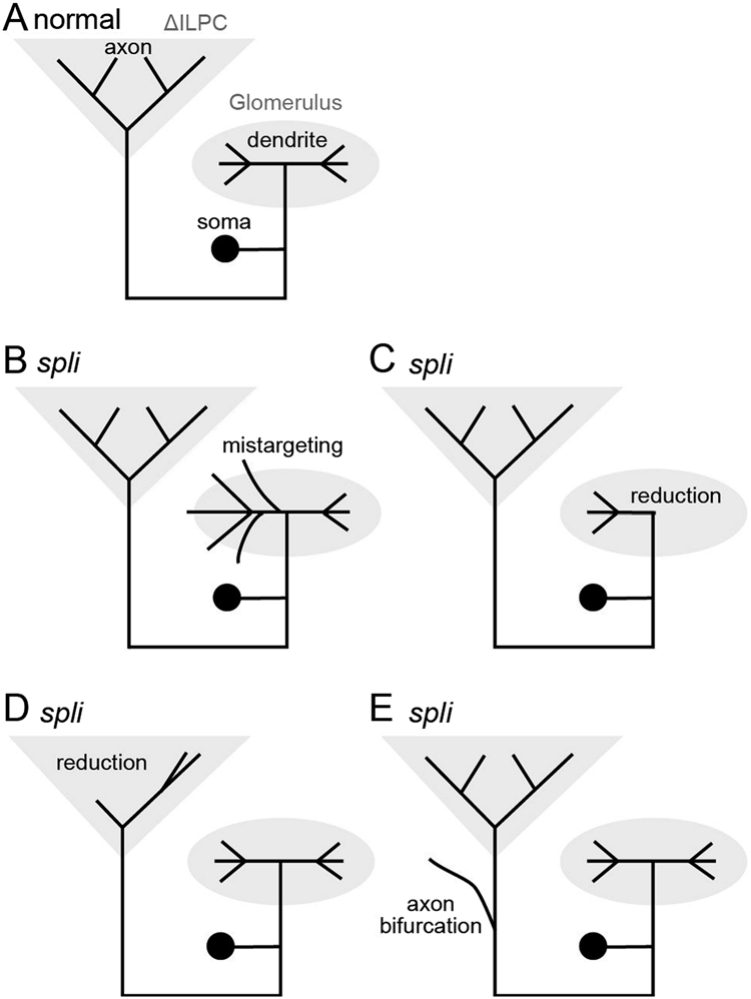
Schematics for the summary of observed change in the morphology of the *spli* mutant. (**A**) Schematics of the morphology of PNs in the silkmoth normal strain. (**B**–**E**) Types of neuroanatomical abnormalities observed in PNs of the *spli* mutant. We observed four abnormal morphological features in the *spli* mutant: PNs usually have a diffuse dendrite that often spills out into areas outside of the glomerulus (**B**); the reduction of dendritic innervation within the glomerulus (**C**); the axonal projection site is altered, and the number of terminals is often reduced (**D**); and the bifurcation of axons (**E**). The axonal bifurcation is not reported in the *acj6*-mutant of *Drosophila*.

We have characterized neuroanatomical abnormalities for the AL PNs in the *spli* mutant of *B. mori* (summarized in Fig. 7). Abnormal morphology is often observed both in the dendrite and axon, suggesting that *Bmacj6* co-ordinately regulates development of these compartments. Contrary to the drastic change in PN morphology, the LN morphology looks indistinguishable from those of the normal strain, suggesting that *Bmacj6* functions in a cell type-specific manner. Because the present study uses the whole-animal mutant (non-mosaic mutant), it is difficult to discriminate whether observed abnormalities were caused by the lack of *Bmacj6* in PNs or cells outside the PNs.

### Mistargeting of dendrite

In this study, we observed extraglomerular innervation of PNs in the AL of the *spli* mutant of *B. mori* (Fig. 7B), which has also been reported in several insect species, including the hawkmoth *Manduca sexta* ^24^ and honeybee *Apis mellifera* ^28,29^. We have previously analysed the presence of the extraglomerular processes for AL PNs in the normal strain of silkmoth^17^. Most of the PNs innervating the ordinary glomeruli (approximately 90%) have extraglomerular processes, which do not enter the other glomeruli but innervate the areas in between the glomeruli^17^. By contrast, only 3% of PNs have extraglomerular processes that innervate the MGC^17^. The extraglomerular processes usually show varicose-like appearance in the normal strain, whereas smooth endings are often observed in the *spli* mutant. These results indicate the presence of an abnormal PN morphology in the *spli* mutant and suggest the function of *Bmacj6* on dendritic targeting.

In one case, we observed a PN innervating both the MGC and an ordinary glomerulus (Fig. 3A). Although a PN innervating the entire AL is present in *Manduca sexta* ^24^, PNs connecting the MGC and a specific ordinary glomerulus have not been reported in moths^24,30–40^, suggesting the abnormality of this morphology.

### Reduced volume in dendrites and axons

We observed a reduction of dendritic and axonal volume of PNs in the *spli* mutant (Fig. 7C,D). The area of innervation of toroid PNs is smaller in the *spli* mutant than in the normal strain (Fig. 3). The reduction of dendritic volume is observed in a specific type of PNs in the *acj6* mutant in *Drosophila* ^14^. Although the glomeruli are always fully innervated by PNs in wildtype, some glomeruli are weakly innervated or not innervated at all in the mutants. Several other transcription factors with similar function are reported^3,14^.

In the PNs innervating the identifiable glomerulus DL1 of *Drosophila acj6* mutants, the total length of the dorsal branch axonal projection within the lateral horn, one of the central targets of PNs, exhibits an eight-fold reduction in length compared with the wildtype dorsal branch^14^. The Kenyon cells, which are downstream neurons of PNs, typically project to both the dorsal and medial lobes of the mushroom body, the higher order centre in the insect brain; however, the Down syndrome cell adhesion molecule (*Dscam)* mutant often fails to send axonal projections to the lobes^41^. Similarly, deficiencies in axonal branching and outgrowth of mushroom body Kenyon cells have been reported in the neuroglian protein mutant^42^.

### Axonal bifurcation

PNs often displayed axonal bifurcation on the AL tract in the *spli* mutant, whereas this morphological feature was not observed in the normal strain (Fig. 7E)^17^. The axonal bifurcation is rarely observed in the PNs of other moth species, including *Manduca sexta* ^24,30–32^, *Spodoptera littoralis* ^33,43^, *Heliothis virescens* ^35–37^, *Helicoverpa assulta* ^38^, *Agrotis ipsilon* ^40^, and *Agrotis segetum* ^39^ (but see Fig. 3C in^44^). This suggests that the observed axonal bifurcation is an abnormal phenotype of the *spli* mutant and that there is a role for *Bmacj6* in the bifurcation of PN axons. Further, we observed that sister branches of the bifurcated axon show mis-targeting (Fig. 6), suggesting a branch-specific control mechanism for axonal-targeting. Axonal bifurcation is not observed in the *acj6* mutant in *Drosophila* ^14^. A possibility is the functional difference of the transcription factor between the species.

The gene controlling axonal bifurcation has been identified in the other cell type in *Drosophila*. DISCO-interacting protein 2 (DIP2) and Glaikit, a downstream target of DIP2, have been identified as factors that regulate axonal bifurcation of mushroom body Kenyon cells^22,45^, the third order neurons in the olfactory pathway. In addition to a failure of axon segregation, the ectopic bifurcation with supernumerary branches has been observed in the DIP2 mutant axon and hence DIP2 acts in Kenyon cells to suppress ectopic branch formation^45^. The function of homolog of the gene in *B. mori* is interesting.

The present study reports the involvement of the transcription factor *Bmacj6* in controlling dendritic targeting and axonal wiring in *B. mori*. We have reported neuroanatomical abnormality in the PNs of the AL in the *Bmacj6* mutant. *Bmacj6* functioning in other neuron types should be addressed in future work.

## Methods

### Experimental animals

The silkmoth *B. mori* strains used were Kinshu-Showa, p50 and w1-pnd. Strain Kinshu-showa and w1-pnd were reared on an artificial diet (SilkMate 2 S and PS; Nosan Corporation Life-Tech Department, Yokohama, Japan), and strain p50 (normal) and n41 with the *spli* gene were reared on fresh mulberry leaves at 26 °C and 60% relative humidity under a long-day photoperiod regime (16/8 hours light/dark). Adult male moths were used 2–7 days after eclosion.

### Intracellular labelling of single neurons

The staining procedure was conducted as previously described^46^. After cooling (4 °C, ∼30 min) to induce anaesthesia, the abdomen, legs, wings, and dorsal side of the thorax were removed. The moth was fixed in a plastic chamber, and its head was immobilized by a notched plastic yoke slipped between the head and thorax. The brain was exposed by opening the head capsule and removing the large tracheae, and the intracranial muscles were removed to eliminate brain movement. The AL was surgically desheathed for insertion of a microelectrode. Filamented glass capillaries (TW100F-3; World Precision Instruments, Sarasota, FL) were pulled on a micropipette puller (P-97 or P-2000; Sutter Instruments, Novato, CA) and filled with 5% Lucifer yellow CH (LY) (Sigma, St. Louis, MO) or 1% tetramethylrhodamine solution (D3308; Molecular Probes, Eugene, OR) in distilled water or 1 M lithium chloride for staining neurons. The resistance of the electrodes was ∼60–300 MΩ. The electrodes were inserted using a micromanipulator (Leica Microsystems, Wetzlar, Germany), and a silver ground electrode was placed on the head cuticle. The brain was superfused with saline solution containing 140 mM NaCl, 5 mM KCl, 7 mM CaCl_2_, 1 mM MgCl_2_, 4 mM NaHCO_3_, 5 mM trehalose, 5 mM N-tris(hydroxymethyl)methyl-2-aminoethanesulfonic acid (TES), and 100 mM sucrose (pH 7.3). We stained each neuron by iontophoretic injection of LY with a constant hyperpolarizing current (approximately −1 to −5 nA) or tetramethylrhodamine with a constant depolarizing current (1 nA) for 1–3 min. After staining, the brain was superfused with saline solution containing 200 mM sucrose. Brains were fixed in 4% paraformaldehyde for 1–8 hours at 4 °C. Brains were then dehydrated with 70%, 80%, 90%, 95%, and 100% ethanol (10 min in each) and cleared in methylsalicylate for at least 30 min.

### Imaging

Each stained neuron was visualized using a confocal imaging system (LSM510; Carl Zeiss, Jena, Germany) with ×40 (numerical aperture = 1.0) objectives. LY-stained neurons were examined at a 458-nm excitation wavelength with a long-pass emission filter (>475 nm) in whole mounts. Tetramethylrhodamine was excited with a HeNe laser at 543 nm, and fluorescence was measured with a 560-nm long-pass filter. Serial optical sections by changing the plane of focus through the thick specimen, were acquired at 0.7-μm intervals throughout the depth of the neuron, and three-dimensional reconstructions of the labelled neurons were created from these sections.

### Data analysis

Segmentation and volume rendering of neurons and neuropils were carried out in AMIRA 6.2 (FEI, Hillsboro, OL). To quantify the dendritic volume, we counted the number of voxels with segmentation data of individual neurons. We excluded soma and axon-like processes for subsequent analysis. The volume distribution of dendrites (Fig. 3D,E) was calculated using a custom-made program written in Matlab and an image processing toolbox (MathWorks, Natick, MA). Maximum intensity projection images were prepared with ImageJ^45^. Figures were prepared in Adobe Illustrator CS (Adobe Systems, San Jose, CA). The datasets generated during and/or analysed during the current study are available from the corresponding author on reasonable request.

## Electronic supplementary material

Supplementary Information
