# Supplementary material for: The morphology of antennal lobe projection neurons is controlled by a POU-domain transcription factor Bmacj6 in the silkmoth Bombyx mori
Source: Sci Rep. 2017 Oct 25;7:14050. doi: 10.1038/s41598-017-14578-4 (PMC5656611; doi:10.1038/s41598-017-14578-4)
Supplement: Supplementary file 1 — Supplementary Information [file 41598_2017_14578_MOESM1_ESM.doc]

**Supplementary Information**

**Article in *Scientific Reports***

**The morphology of antennal lobe projection neurons is controlled by a POU-domain transcription factor Bmacj6 in the silkmoth *Bombyx mori***

Shigehiro Namiki, Tsuguru Fujii, Toru Shimada & Ryohei Kanzaki


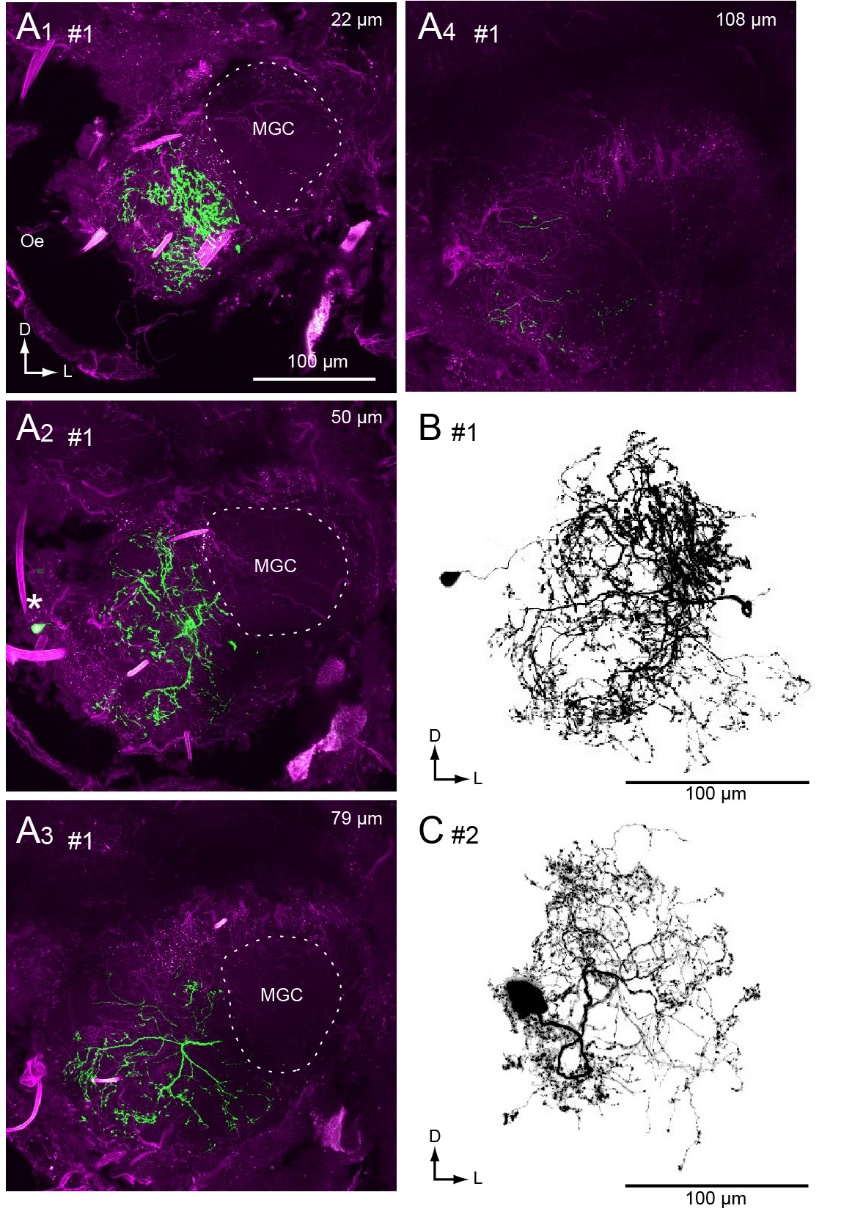


**Supplementary Figure 1. Morphology of antennal lobe local interneurons in the *spli* mutant.** (**A**) Confocal microscopic images at four different depths are shown. The depth from the anterior surface is shown in the *top right*. The cell body position is marked by an asterisk (A2). (**B, C**) Maximum intensity projection of the identified local interneurons. We have stained total two local interneurons.
